# Mid-Season Influenza Virus Genetic Characterizations in U.S. Department of War Populations, 2025–2026

**Published:** 2026-08-20

**Authors:** William E. Gruner, Deanna Muehleman, Vivian S. Hogan, Daniel F. Ewing, Appavu K. Sundaram, Anthony C. Fries

**Affiliations:** Defense Centers for Public Health, Wright-Patterson Air Force Base, Dayton, OH; JYG Innovations, LLC, Dayton; U.S. Air Force School of Aerospace Medicine, Wright-Patterson Air Force Base; Naval Medical Research Command, Ft. Detrick, Frederick, MD

## Abstract

Continuous monitoring of influenza antigenic drift is vital for vaccine strain selection. This study utilized global Department of War (DOW) surveillance data to evaluate circulating influenza strains and inform the 2026-2027 Northern Hemisphere influenza vaccine recommendations by the World Health Organization. From September 2025 through February 2026, the Department of Defense Global Respiratory Pathogen Surveillance Program collected and analyzed respiratory specimens from over 100 sentinel sites globally. Influenza-positive samples underwent next-generation sequencing, phylogenetic analysis, and antigenic characterization. Analysis of 1,300 sequences revealed A(H3N2) as the predominant (80.1%) subtype, followed by A(H1N1)pdm09 (10.6%) and B/Victoria (9.3%), with variations between combatant commands. Significant antigenic drift led to the dominance of subclades K, D.3.1/D.3.1.1, and C.3.1, respectively. Testing indicated that the 2025-2026 vaccine components offered reduced protection against these emerging strains. Antigenic cartography confirms that the 2026-2027 vaccine strains provide significantly improved protection against circulating viruses. Antiviral resistance markers for neuraminidase inhibitors such as oseltamivir remain rare, with only 2 identified in A(H1N1)pdm09. These data are critical for maintaining force health protection and assessing vaccine performance within highly mobile DOW populations.

Annual evaluation and updates, as necessary, of seasonal influenza vaccine strains remain important to counter constant antigenic drift. ^1^ The U.S. Department of War (DOW), with a historically highly vaccinated, global population, produces critical surveillance data that help inform annual influenza strain selections. ^2^ Data collected from the Department of Defense Global Respiratory Pathogen Surveillance Program (DoDGRPSP) ^3^ and Global Emerging Infections Surveillance (GEIS)-funded global partner laboratories provide a robust surveillance picture for monitoring influenza activity and antigenic drift throughout the year. ^4^

On February 27, 2026, the World Health Organization (WHO) recommended updates for the 2026-2027 Northern Hemisphere influenza vaccine: transitioning the A(H1N1)pdm09 component to a subclade D.3.1 A/Missouri/11/2025-like virus for all vaccine platforms (egg, cell, recombinant), changing the A(H3N2) component to a subclade K A/Darwin/1454/2025-like virus (egg) and A/Darwin/1415/2025-like virus (cell/recombinant), and the B/Victoria component to a subclade C.3.1 B/Tokyo/EIS13-175/2025-like virus (egg) or B/Pennsylvania/14/2025-like virus (cell/recombinant). ^5^ This report utilizes DOW genetic, antigenic, and epidemiological data, presented to the U.S. Food and Drug Administration (FDA)’s Vaccines and Related Biological Products Advisory Committee (VRBPAC) on March 12, 202'6, to contextualize the new WHO recommendations. ^6^

**What are the new findings?**

Analysis of 2025-2026 influenza season data revealed the predominance of subclades A(H1N1)pdm09 D.3.1/D.3.1.1, A(H3N2) K, and B/Victoria C.3.1. Antigenic cartography employing recent candidate vaccine viruses from the U.S. Centers for Disease Control and Prevention confirmed that the World Health Organization’s strain recommendations for the 2026-2027 Northern Hemisphere influenza vaccine provide the best protection against these circulating strains.

**What is the impact on readiness and force health protection?**

Influenza illnesses directly reduce military force readiness. Annual vaccination remains a proven mechanism for reducing cases and mitigating their severity. These data from the Department of War, presented at the 2026 U.S. Food and Drug Administration Vaccines and Related Biological Products Advisory Committee meeting, in conjunction with U.S. Centers for Disease Control and Prevention findings, informed the selection of the most appropriate virus strains for the 2026-2027 influenza vaccine, to optimize protection for U.S. service members.

## Methods

Influenza data from the DOW were accumulated from analyses of respiratory specimens collected as part of DoDGRPSP systematic sampling from more than 100 sentinel sites, along with sequence data contributed from GEIS-funded partner laboratories around the world, from September 1, 2025 through February 17, 2026. Specimens collected through DoDGRPSP efforts were first tested using a commercial real-time polymerase chain reaction (PCR) multiplex pathogen panel to determine influenza positivity and type or subtype. Regional differences in subtype proportions were assessed using Pearson’s chi-squared test in R Statistical Software (version 4.2.3). Test-positive influenza specimens were subsequently sequenced using Illumina next-generation sequencing technology and analyzed using Nextstrain to determine clade and subclade. Other analytical tools were used to construct phylogenetic trees and screen amino acid substitutions consistent with changes to hemagglutinin (HA) glycosylation motifs and neuraminidase (NA) markers of resistance to NA-inhibiting antiviral drugs such as oseltamivir. Frequently occurring substitutions that were in addition to substitutions consistent with subclade designations **(Table 1)** were also noted. A subset of positive influenza specimens that were genetically characterized were also sent to partners at the Naval Medical Research Command (NMRC) for antigenic characterization using the High-content Imaging-based micro-Neutralization Test (HINT) and antigenic cartography mapping. ^7 - 8^ Specimens sent for HINT testing were selected based on distribution across collection dates and location as well as clade and subclade diversity.

**TABLE 1.** Distribution of Influenza Subclade Diversity, by Influenza A Subtype and B/Victoria Lineage Virus, U.S. Department of War, 2025–2026

Subtype, Lineage	Clade	Subclade	No.	% of Total Subtype	Defining Substitutions ^a^
A(H1N1)pdm09	5a.2a	C.1.9	3	2.2	K169Q
		C.1.9.3	7	5.1	S83P, K169Q
	5a.2a.1	D.3.1 ^b^	72	52.2	I372V, I460T, V520A
		D.3.1.1	56	40.6	R113K, A139D, E283K, K302E
A(H3N2)	2a.3a.1	J.2 ^c^	14	1.3	K276E
		J.2.2	6	4.3	T65K, S124N
		J.2.3	7	0.7	S378N
		J.2.4	39	3.7	T135K
		K ^d^	975	93.7	K2N, Q173R
B/Victoria	V1A.3a.2	C.3	16	13.2	A154E, S208P, S255P
		C.3.1 ^e^	87	71.9	D197N
		C.5.1	2	1.7	E128K, E183K
		C.5.6	11	9.1	D128E
		C.5.6.1	4	3.3	T37I, P241Q
		C.5.7	1	0.8	E128G, E183K

Abbreviation: No., number.

a All substitutions reference changes in hemagglutinin subunit 1 (HA1).

b Subclade of selected 2026-2027 Northern Hemisphere A(H1N1)pdm09 strain A/Missouri/11/2025 (egg, cell).

c Subclade of previous 2025-2026 Northern Hemisphere A(H3N2) strains A/Croatia/10136RV/2023 (egg) and A/District of Columbia/27/2023 (cell).

d Subclade of selected 2026-2027 Northern Hemisphere A(H3N2) strains A/Darwin/1454/2025 (egg) and A/Darwin/1415/2025 (cell).

e Subclade of selected 2026-2027 Northern Hemisphere B/Victoria strains B/Tokyo/EIS13-175/2025 (egg) and B/Pennsylvania/14/2025 (cell).

## Results

DOW data comprised 1,300 influenza sequences in total, including data for 138 A(H1N1)pdm09 (10.6%), 1,041 A(H3N2) (80.1%), and 121 B/Victoria (9.3%). Although A(H3N2) was the predominant subtype circulating throughout the sentinel site network, some regional differences were observed **(Figure 1)** . A higher proportion of A(H1N1)pdm09 was observed in U.S. European Command (EUCOM), West Africa, Hawai'i, and U.S. Department of Health and Human Services (HHS) regions 3, 5, and 9 than in other geographic locations, while a higher proportion of B/Victoria was observed in South Korea, East Africa, and HHS regions 4, 6, 7, and 10 than in other geographic locations. Examining DOW subtype by combatant command (CCMD), statistically significant differences included higher than expected A(H1N1)pdm09 in EUCOM and B/Victoria in U.S. Northern Command (NORTHCOM), and lower than expected A(H1N1)pdm09 in NORTHCOM, B/Victoria in U.S. Africa Command (AFRICOM), and B/Victoria in EUCOM. These dynamics are consistent with CDC and WHO data that showed regional variation of subtype diversity across continents. ^5^

**FIGURE 1.**
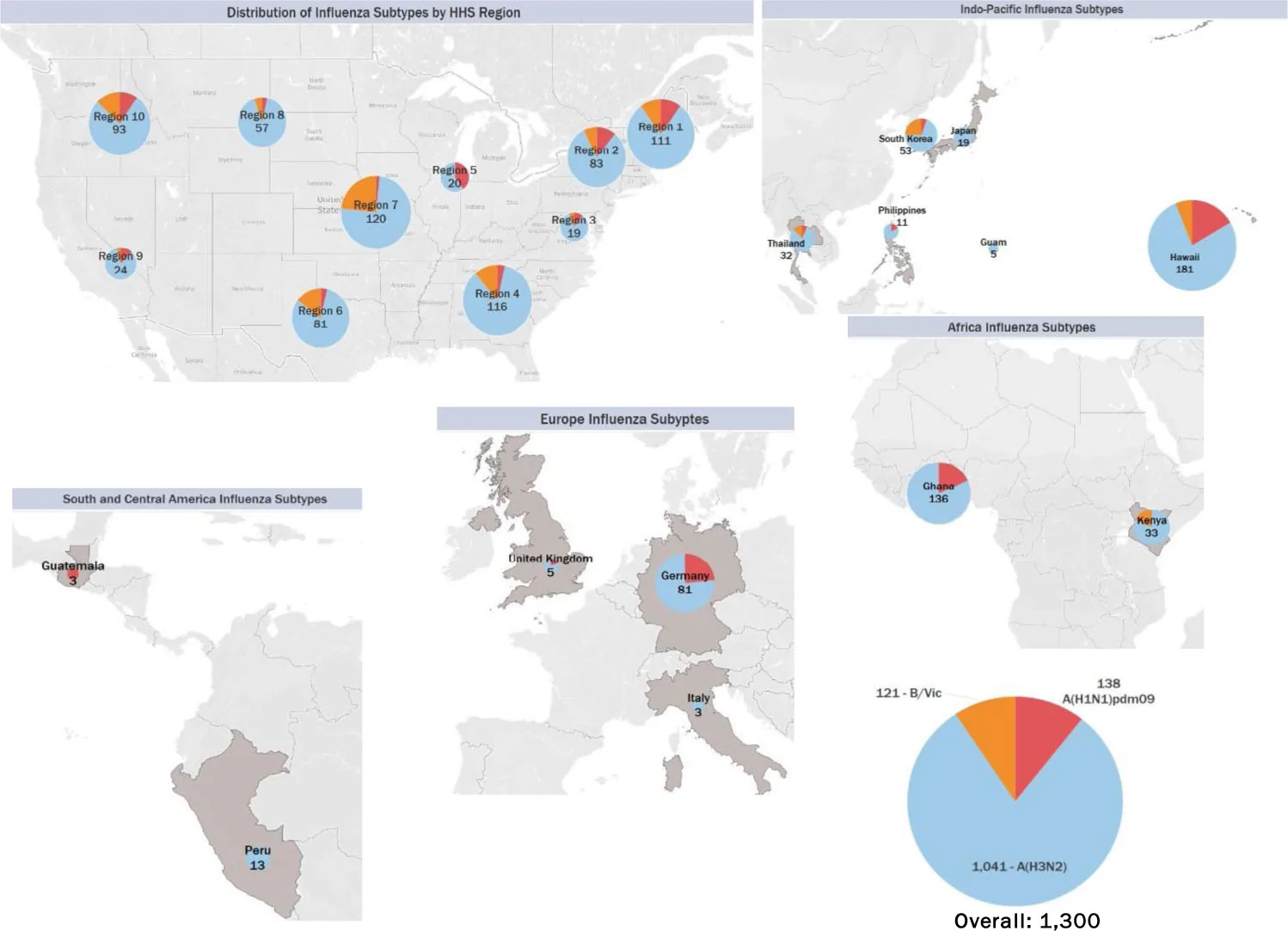
Geographic Distribution of Influenza Phylogenetic Analysis Sequence Data

Phylogenetic analysis of the DOW A(H1N1)pdm09 HA data revealed an approximately even split between circulating subclades D.3.1 and D.3.1.1, at 52.2% and 40.6% of sequences, respectively, while the 2024-2025 predominant subclades C.1.9 and C.1.9.3 continued to co-circulate at 2.2% and 5.1%, respectively **(Table 1** , **Supplementary Figure 1a)** . The Northern Hemisphere 2026-2027 WHO influenza vaccine strain recommendation for both egg and cell/recombinant formulations, A/Missouri/11/2025-like viruses, sat firmly in the middle of the branching for D.3.1 **(Figure 2a)** . Incorporating HINT-based antigenic characterization performed at NMRC and utilizing post-infection ferret antisera raised against vaccine candidate strains, 2 subclade D.3.1 viruses out of 23 (8.7%) DOW A(H1N1)pdm09 viruses tested showed low reaction, as defined as a greater than 4-fold decrease in titer relative to the homologous titer for A/Wisconsin/67/2022-like virus (Northern Hemisphere 2025-2026 strain component). These 2 viruses had HA1 amino acid substitutions of either R205K or S157M and D168N. One (4.3%) additional subclade D.3.1 virus with the HA1 substitution I510V showed a greater than 4-fold decrease in titer relative to homologous titers for both A/Wisconsin/67/2022-like and A/Missouri/11/2025-like virus post-infection ferret antisera (red bars to right, **Figure 2a** ). High reacting strains, as defined as a less than 4-fold decrease in titer relative to the homologous titer, are represented as yellow bars to the right of the phylogenetic trees. Antigenic cartography confirmed that the updated Northern Hemisphere vaccine strain recommendation, A/Missouri/11/2025-like virus, shifts protection toward the predominantly circulating D.3.1 and D.3.1.1 strains compared to the 2025-2026 vaccine component **(Figure 3a** , **Supplementary Table 1a)** .

**FIGURE 2a.**
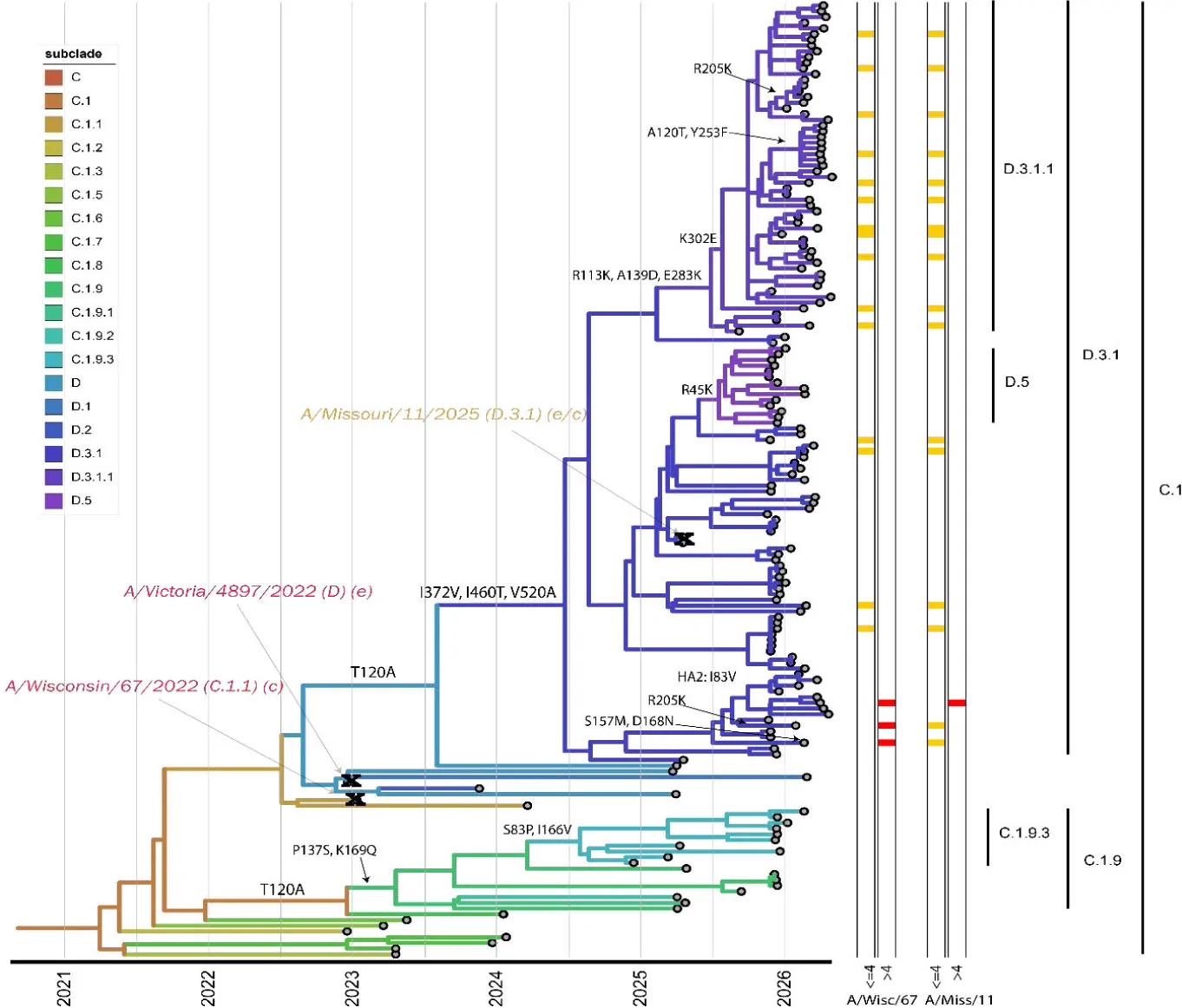
Influenza A(H1N1)pdm09 Hemagglutinin Coding Sequence Phylogenetic Tree, U.S. Department of War, 2025–2026

**FIGURE 3a.**
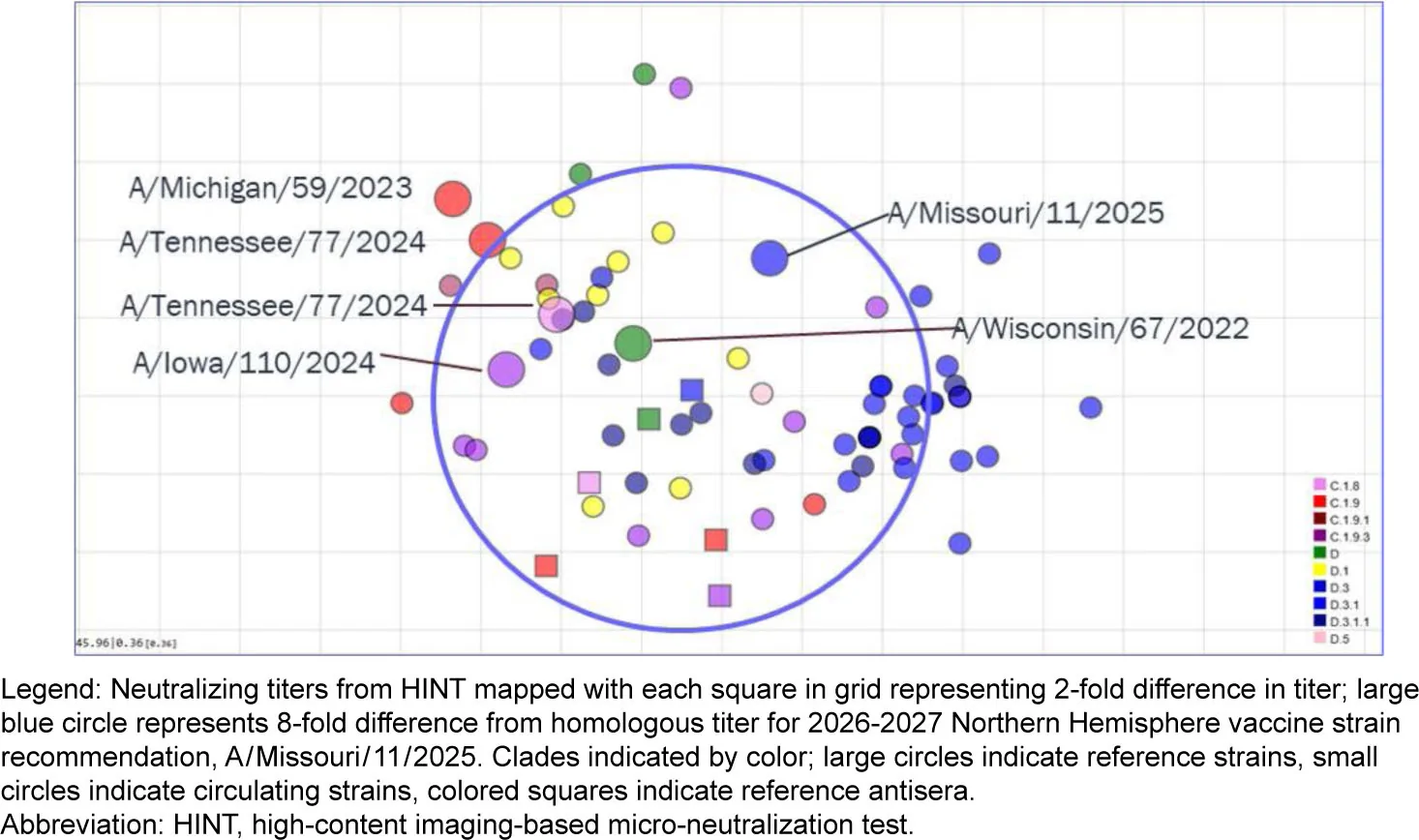
Influenza A(H1N1)pdm09 Antigenic Cartography Map

No changes to glycosylation sites, which can alter immune response, were observed for the A(H1N1)pdm09 viruses characterized. One virus contained the NA substitution H275Y in the consensus sequence, while another contained I223V and S247N, both of which may confer NA inhibitor antiviral resistance. ^9^

Phylogenetic analysis of the DOW A(H3N2) HA data showed that subclade K predominated, overwhelmingly, contributing 93.7% of the circulating viruses sequenced, while subclades J.2, J.2.2, J.2.3, and J.2.4 co-circulated at very low levels **(Table 1** , **Supplementary Figure 1b)** . HINT data using post-infection ferret antisera raised against A/District of Columbia/27/2023-like virus (Northern Hemisphere 2025-26 cell-component) showed 36 out of 144 (25.0%) representative viruses with a greater than 4-fold decrease from the homologous titer, whereas only 20 (13.9%) viruses showed a greater than 4-fold decrease in titer to A/Wisconsin/114/2025, an A/Darwin/1415/2025-like virus (red bars, **Figure 2b** ). Antigenic cartography confirmed that the Northern Hemisphere 2026-2027 recommendations shift protection towards the predominantly circulating subclade K strains as compared to the Northern Hemisphere 2025-2026 vaccine components **(Figure 3b** , **Supplementary Table 1b** . All subclade J.2.4 and K sequences shared the loss of a glycosylation site due to the HA1 amino acid substitution at T135K, while all the subclade K and many of the J.2.4 sequences shared the gain of a glycosylation site due to the HA1 amino acid substitution S144N **(Figure 2b)** . No consensus NA sequences showed markers for antiviral resistance.

**FIGURE 2b.**
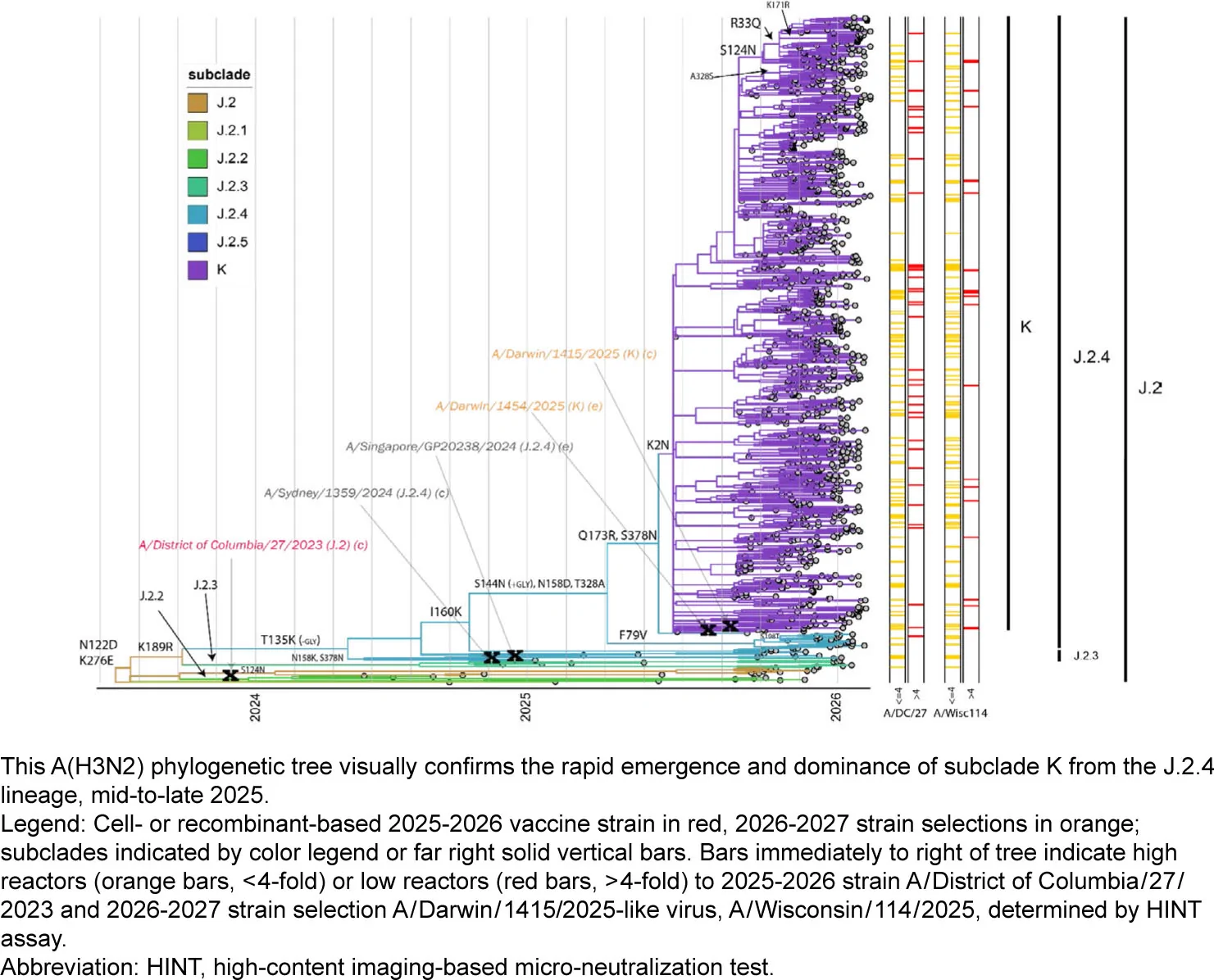
Influenza A(H3N2) Hemagglutinin Coding Sequence Phylogenetic Tree, U.S. Department of War, 2025–2026

**FIGURE 3b.**
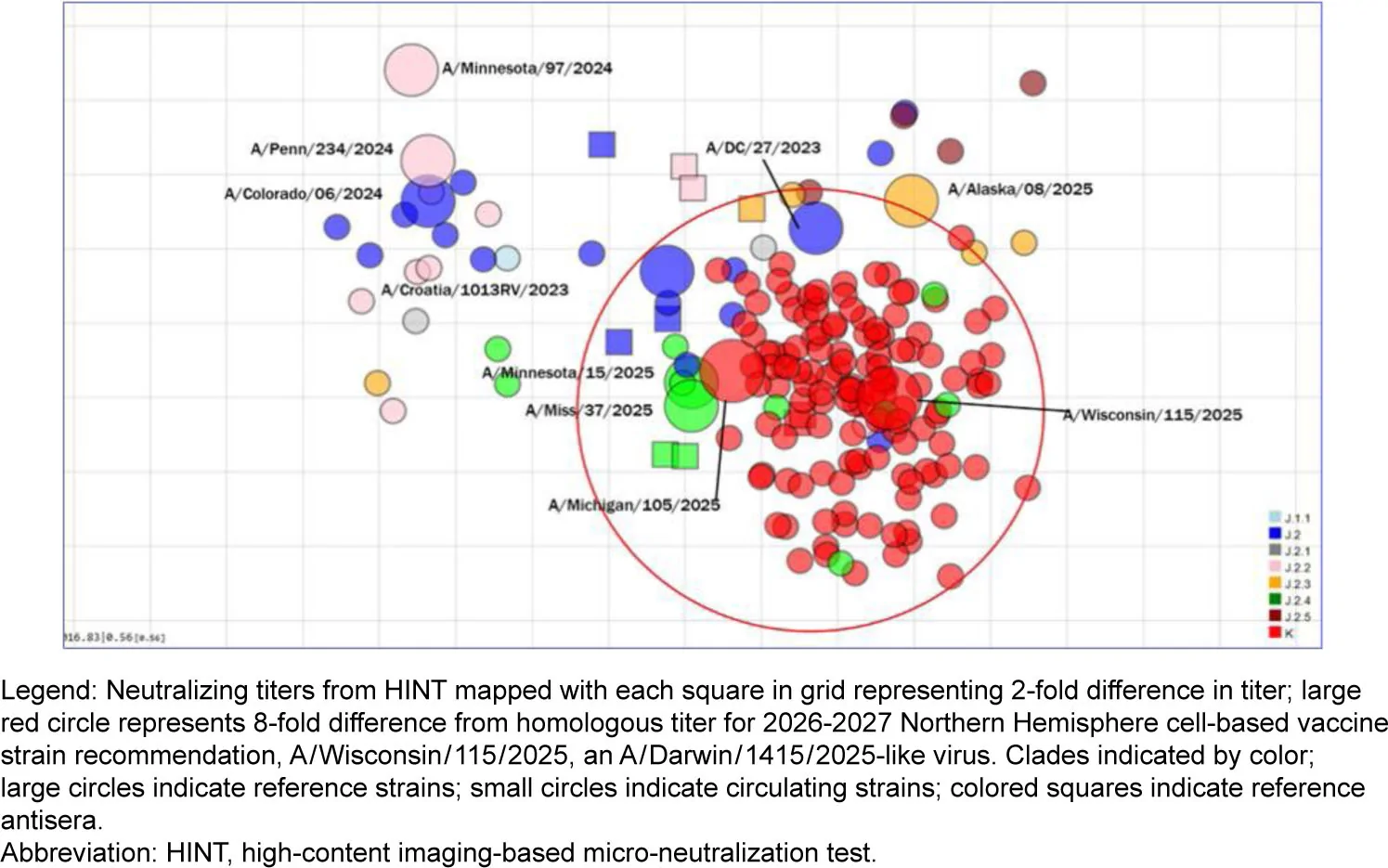
Influenza A(H3N2) Antigenic Cartography Map

Phylogenetic analysis of DOW B/Victoria data showed that 71.9% of the HA sequences belonged to the predominantly circulating subclade C.3.1, while moderate amounts of subclades C.3 and C.5.6 co-circulated at 13.2% and 9.1%, respectively, and subclades C.5.1, C.5.6.1, and C.5.7 co-circulated at lower levels **(Table 1** , **Supplementary Figure 1c)** . The Northern Hemisphere 2026-2027 WHO vaccine strain recommendation is B/Tokyo/EIS13-175/2025-like (egg) and B/Pennsylvania/14/2025-like (cell/recombinant) viruses from subclade C.3.1. HINT data using post-infection ferret antisera raised against the Northern Hemisphere 2025-2026 component, B/Austria/1359417/2021-like virus, showed 13 out of 18 (72.2%) representative viruses with greater than a 4-fold decrease from the homologous titer (red bars), but no low reactors to B/Pennsylvania/14/2025 were observed **(Figure 2c)** . Antigenic cartography data indicate distinct clustering of C.3.1 viruses away from the previous B/Austria/1359417/2021-like vaccine strain, justifying the recommended update **(Figure 3c** , **Supplementary Table 1c)** . All subclade C.3.1 sequences and most C.3 sequences shared the HA1 amino acid substitution D197N, which caused the addition of a glycosylation site **(Figure 2c)** . Again, no consensus NA sequences showed markers for antiviral resistance.

**FIGURE 2c.**
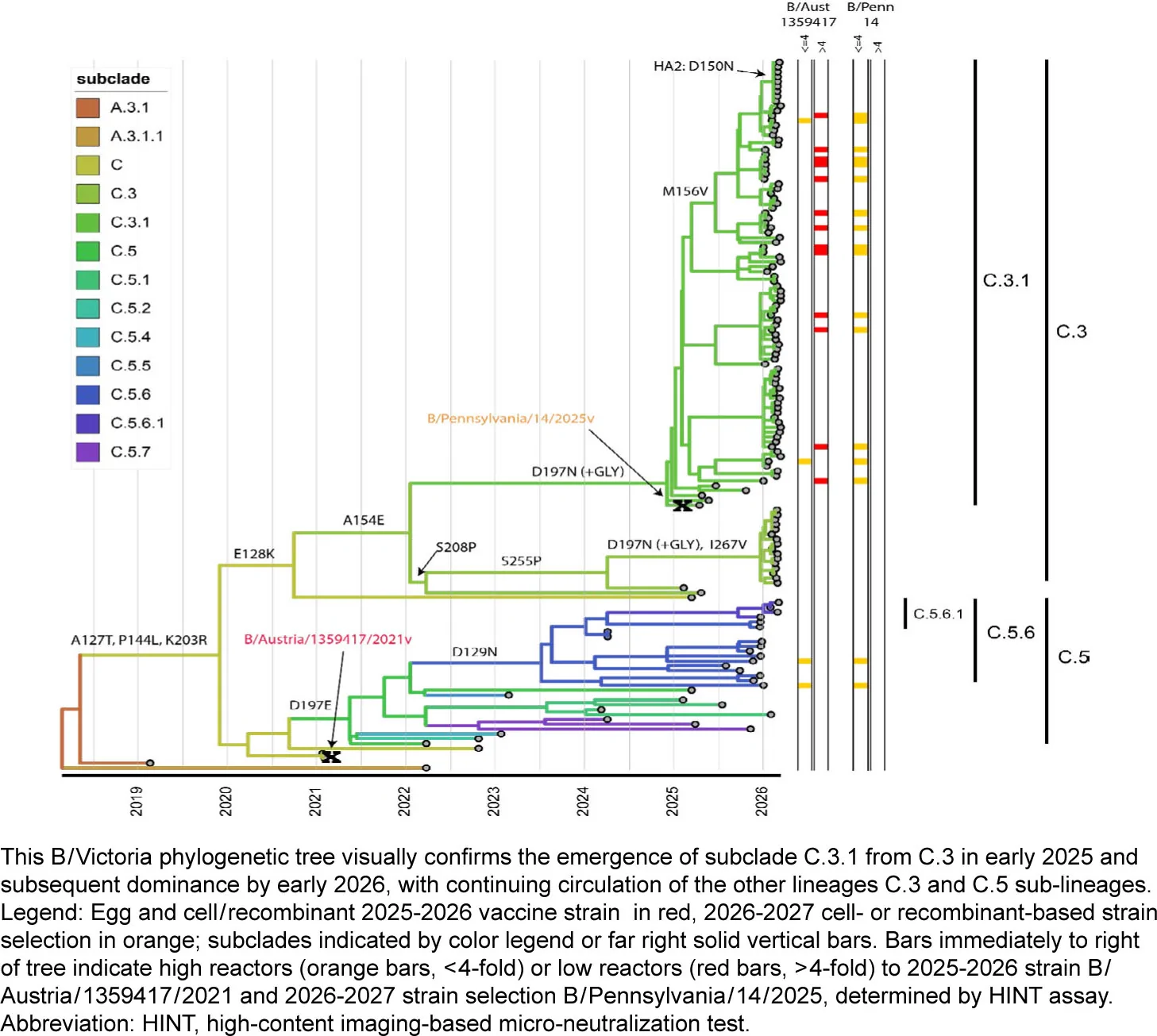
Influenza B/Victoria Hemagglutinin Coding Sequence Phylogenetic Tree, U.S. Department of War, 2025–2026

**FIGURE 3c.**
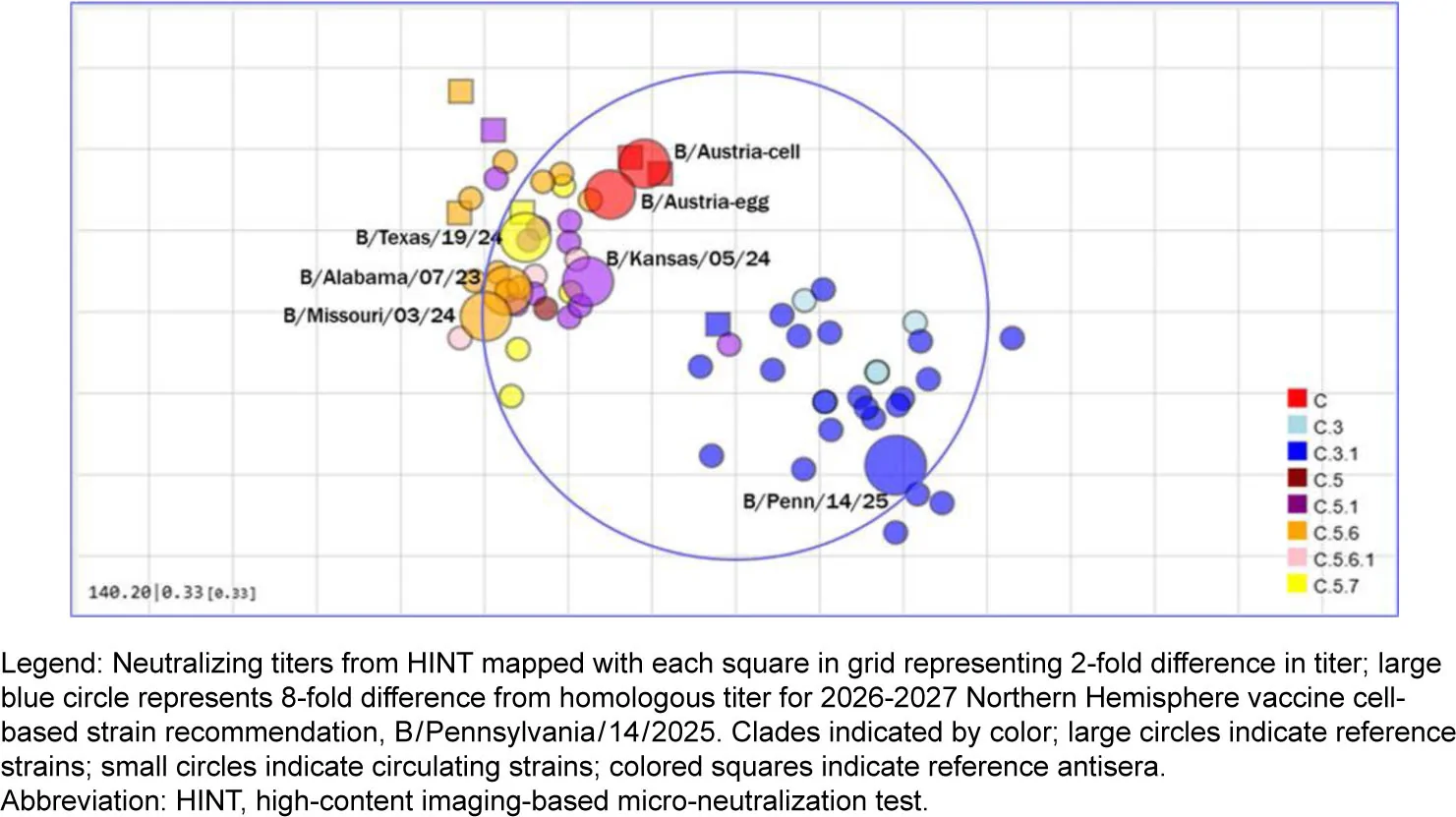
Influenza B/Victoria Antigenic Cartography Map

## Discussion

Analysis of the DOW influenza sequence data collected by GEIS partner laboratories and presented at the 2026 VRBPAC meeting, in combination with antigenic characterization data, support the WHO recommendations for updating the A(H1N1)pdm09, A(H3N2), and B/Victoria strain components for the Northern Hemisphere 2026-2027 season. In conjunction with mid-season vaccine data, these analyses complement national CDC data and remain essential for assessing vaccine performance in the highly mobile, global DOW populations.

Antigenic drift was observed throughout the 2025-2026 season for influenza A(H1N1)pdm09, A(H3N2), and B/Victoria, leading to majority strain circulation in the subclades D.3.1.1, K, and C.3.1, respectively. The 2025-2026 Northern Hemisphere influenza vaccine strains show evidence of reduced protection against those strains, whereas the WHO strain recommendations for the 2026-2027 vaccine demonstrate improved protection against tested representatives from those subclades. A(H3N2) was the most prominent circulating subtype in global CCMDs, but A(H1N1)pdm09 and B/Victoria displayed more circulating subclade diversity than A(H3N2).

While annual influenza vaccination remains a proven method of protection against influenza disease burden, antiviral treatment such as the NA inhibitor oseltamivir is an effective method to mitigate influenza case severity. Two A(H1N1)pdm09 consensus sequences analyzed in this study displayed molecular markers in the NA gene that may confer drug resistance. Continued monitoring of substitutions that confer resistance, along with substitutions that result in loss or gain of glycosylation motifs that may affect virus receptor binding and virulence, are important to interpret in addition to estimates of influenza burden in the DOW. ^10^

Limitations of this study include uneven sentinel site participation and lack of sampling sites in some global regions, resulting in incomplete representation of global influenza circulation. Additionally, detection of antiviral resistance markers relying solely on consensus sequencing data is not definitive; subsequent studies will include investigation of minor variants as well as phenotypic assays for confirmation.
