# Supplementary material for: Mid-Season Influenza Virus Genetic Characterizations in U.S. Department of War Populations, 2025–2026
Source: MSMR. 2026 Aug 20;33(8):32–41. (PMC13645439)
Supplement: Supplementary file 1 [file msmr-33-8-32-s01.pdf]

SUPPLEMENTARY FIGURE 1a. Influenza A(H1N1)pdm09 Specimen Numbers and Subclade Proportions, September 2025–February 2026

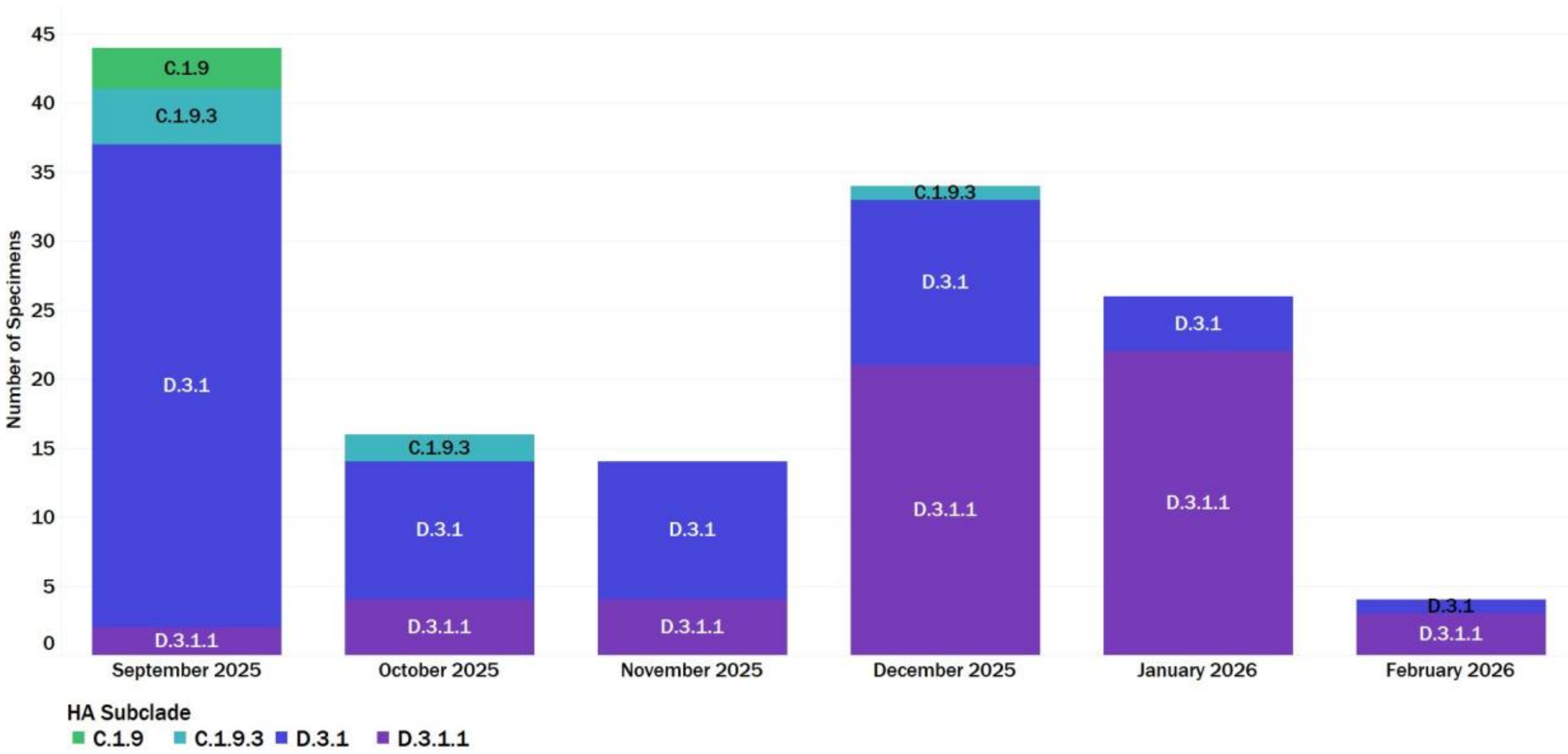

Abbreviation: HA, hemagglutinin.

SUPPLEMENTARY FIGURE 1b. Influenza A(H3N2) Specimen Numbers and Subclade Proportions, September 2025–February 2026

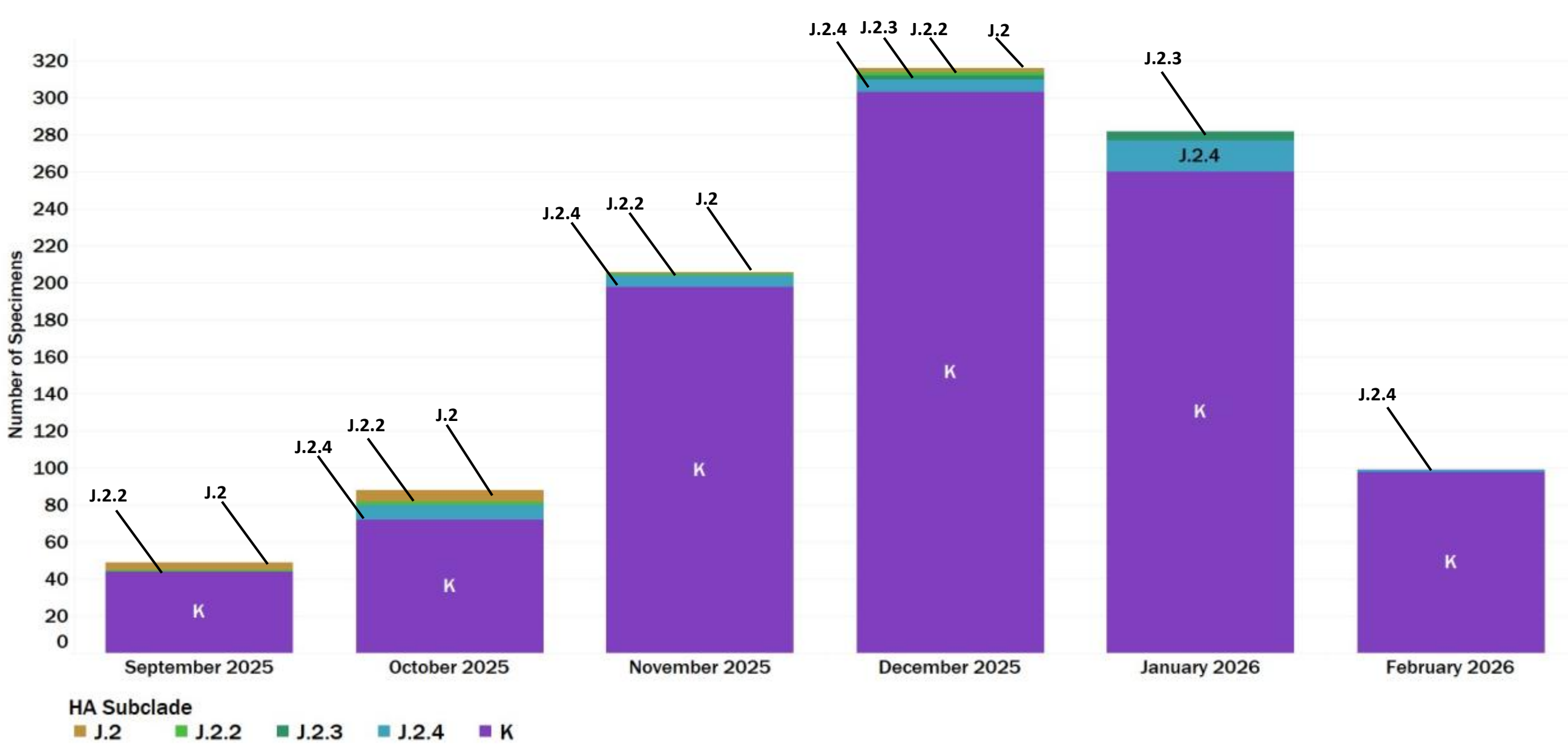

Abbreviation: HA, hemagglutinin.

SUPPLEMENTARY FIGURE 1c. Influenza B/Victoria Specimen Numbers and Subclade Proportions, September 2025–February 2026

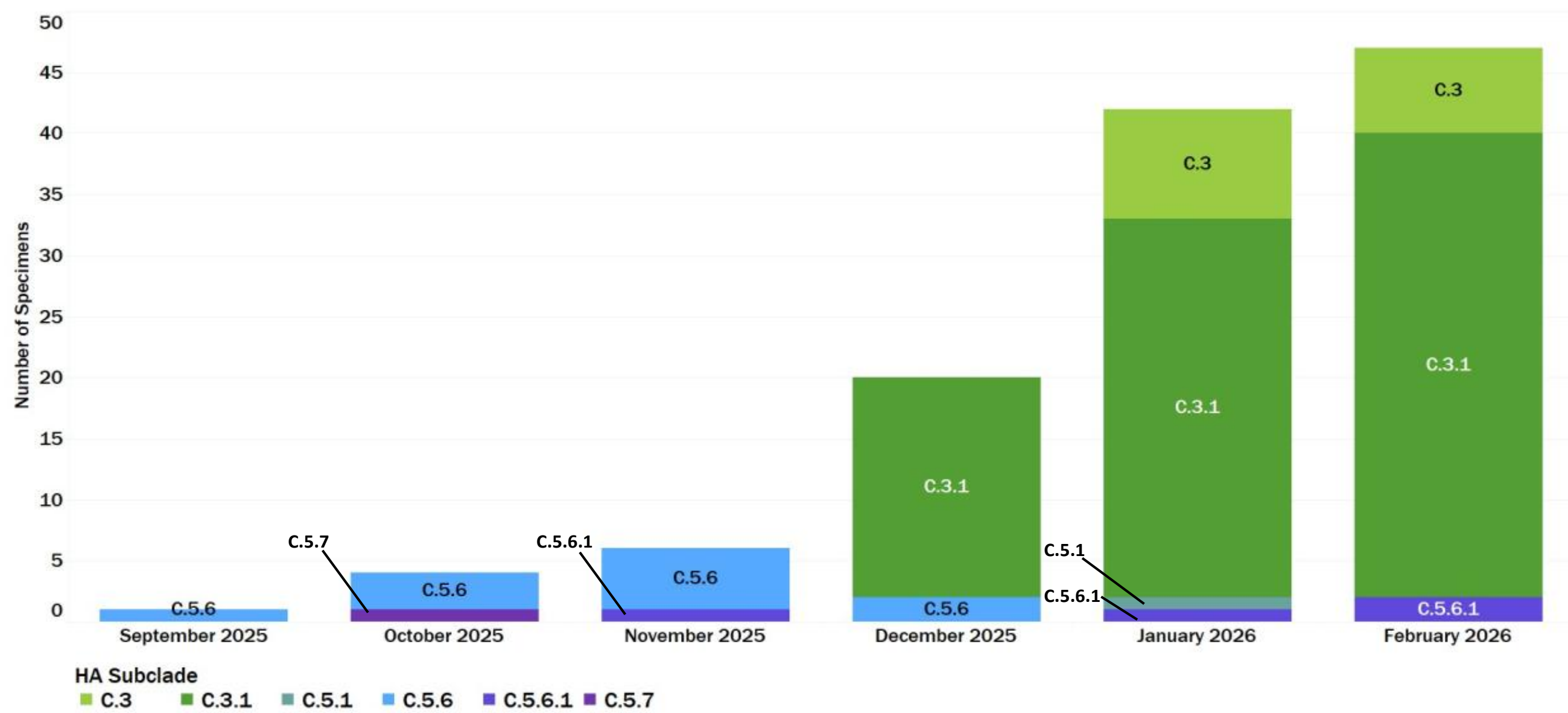

Abbreviation: HA, hemagglutinin.

SUPPLEMENTARY TABLE 1a. Influenza A(H1N1)pdm09 HINT Assay Antigenic Characterization Titer Table

Viruses Tested	Clade	Reference Antisera A/WI/67/22 c[a] C.1.1	A/Vic/4897/22 e[a] D	A/CT/17/23 C.1.8	A/MI/59/23 C.1.9	A/IA/110/24 C.1.9.3	A/MO/11/25 c[b] D.3.1	A/MO/11/25 e[b] D.3.1
**Reference virus**								
A/Wisconsin/67/2022 c[a]	C.1.1	**2,560[c]**	1,280	1,280	1,280	640	1,280	640
A/Victoria/4897/2022 e[a]	D	640	**320[c]**	320	320	80	320	160
A/Connecticut/17/2023	C.1.8	2,560	640	**1,280[c]**	640	320	320	320
A/Michigan/59/2023	C.1.9	320	160	320	**640[c]**	160	320	320
A/Iowa/110/2024	C.1.9.3	640	320	1,280	1,280	**640[c]**	640	640
A/Missouri/11/2025 c[b]	D.3.1	640	640	640	320	320	**640[c]**	1,280
A/Missouri/11/2025 e[b]	D.3.1	10,240	2,560	5,120	5,120	1,280	640	**640[c]**
**Test virus**								
H125062	C.1.9	640	160	640	1,280	160	160	80
H125069	C.1.9	1,280	640	1,280	1,280	1,280	320	1,280
H125065	C.1.9.1	320	320	640	640	160	320	160
H125066	C.1.9.1	640	320	1,280	640	320	640	640
H125073	C.1.9.3	1,280	320	640	2,560	1,280	640	640
H125092	C.1.9.3	640	320	320	640	640	320	640
H125107	C.1.9.3	1,280	2,560	1,280	1,280	640	640	640
H125063	D	640	320	320	320	80	320	320
H125074	D	320	160	80	160	40	320	160
H125081	D.1	1,280	1,280	640	1,280	160	1,280	320
H125064	D.3.1	1,280	640	640	320	160	640	320
H125100	D.3.1	640	1,280	320	320	640	640	640
H125103	D.3.1	320	1,280	160	160	320	320	640
H125106	D.3.1	640	2,560	320	640	1,280	1,280	2,560
H126002	D.3.1	640	1,280	320	320	640	640	1,280
H126003	D.3.1.1	640	1,280	640	320	1,280	640	1,280
H126019	D.3.1.1	2,560	2,560	1,280	2,560	1,280	2,560	2,560
H126021	D.3.1.1	1,280	1,280	1,280	1,280	320	1,280	1,280
H125070	D.5	1,280	320	1,280	1,280	640	1,280	1,280
H125095	D.5	320	320	160	160	320	320	1,280

Abbreviations: HINT, high-content imaging-based micro-neutralization test; WI, Wisconsin; Vic, Victoria; CT, Connecticut; MI, Michigan; IA, Iowa; MO, Missouri.
[a] Titers against 2025-2026 vaccine strain selections.
[b] Titers against 2026-2027 vaccine strain recommendations.
[c] Homologous titers.

SUPPLEMENTARY TABLE 1b. Influenza A(H3N2) HINT Assay Antigenic Characterization Titer Table

Viruses Tested	Clade	Reference Antisera							
		A/Cro/10136RV/23 e[a]	A/DC/27/23 c[a]	A/CO/06/24	A/PA/234/24	A/AK/08/25	A/MN/15/25	A/MI/105/25 e[b]	A/WI/114/25 c[b]
	Clade	J.2	J.2	J.2.1	J.2.2	J.2.3	J.2.4	K	K
**Reference virus**									
A/DC/27/2023 c[a]	J.2	**10,240[c]**	5,120	2,560	5,120	2,560	5,120	5,120	5,120
A/Croatia/10136RV/2023 e[a]	J.2	5,120	**1,280[c]**	640	1,280	640	1,280	2,560	2,560
A/Colorado/06/2024	J.2.1	2,560	640	**640[c]**	320	320	640	320	320
A/Pennsylvania/234/2024	J.2.2	1,280	320	640	**320[c]**	320	320	320	640
A/Alaska/08/2025	J.2.3	1,280	320	320	320	**1,280[c]**	1,280	1,280	1,280
A/Minnesota/15/2025	J.2.4	5,120	2,560	640	640	320	**5,120[c]**	10,240	10,240
A/Michigan/105/2025[b]	K	10,240	2,560	160	1,280	1,280	10,240	**20,480[c]**	20,480
A/Wisconsin/114/2025[b]	K	1,280	320	80	320	640	2,560	10,240	**10,240[c]**
**Test virus**									
H325085	J.1.1	2,560	640	320	1280	640	1,280	1,280	2,560
H325064	J.2	5,120	1,280	640	1,280	1,280	2,560	2,560	2,560
H325075	J.2	2,560	640	80	160	640	5,120	5,120	5,120
H325103	J.2	2,560	1,280	640	640	640	1,280	640	1,280
H325107	J.2	1,280	640	640	640	320	640	640	640
H325071	J.2.1	1,280	640	640	320	80	1,280	640	1,280
H325072	J.2.1	5,120	640	640	2,560	2,560	2,560	2,560	5,120
H325066	J.2.2	1,280	320	640	640	160	640	640	640
H325086	J.2.2	1,280	640	160	160	80	640	640	1,280
H325089	J.2.2	1,280	320	160	160	160	320	320	1,280
H325104	J.2.2	2,560	640	640	640	640	640	640	1,280
H325084	J.2.3	640	160	80	80	1,280	1,280	320	640
H325088	J.2.3	1,280	320	80	160	1,280	1,280	1,280	1,280
H326082	J.2.3	2,560	640	320	1,280	2,560	2,560	1,280	5,120
H326071	J.2.4	640	160	80	640	1,280	2,560	5,120	2,560
H326088	J.2.4	2,560	1,280	80	320	640	2,560	2,560	2,560
H326128	J.2.4	1,280	320	80	160	320	1,280	5,120	2,560
H326131	J.2.4	640	320	20	40	320	5,120	5,120	5,120
H325077	J.2.5	320	160	80	80	640	160	320	640
H325079	J.2.5	2,560	160	160	320	2,560	640	640	640
H325083	J.2.5	1,280	160	160	160	2,560	1,280	640	640
H325106	J.2.5	2,560	1,280	640	1,280	2,560	1,280	1,280	2,560
H325094	K	1,280	320	320	640	2,560	2,560	5,120	10,240
H325095	K	320	320	160	640	640	2,560	5,120	5,120
H326006	K	1,280	640	80	640	640	5,120	10,240	10,240
H326117	K	640	640	80	160	320	640	1,280	10,240
H326118	K	640	320	80	160	640	1,280	5,120	2,560
H326130	K	1,280	640	160	640	1,280	2,560	10,240	5,120

Abbreviations: HINT, high-content imaging-based micro-neutralization test; Cro, Croatia; DC, District of Columbia; CO, Colorado; PA, Pennsylvania; AK, Alaska; MN, Minnesota; MI, Michigan; WI, Wisconsin.
[a] Titers against 2025-2026 vaccine strain selections.
[b] Titers against 2026-2027 vaccine strain recommendations.
[c] Homologous titers.
